# Perovskite Solar Cells Based on Compact, Smooth FA_0.1_MA_0.9_PbI_3_ Film with Efficiency Exceeding 22%

**DOI:** 10.1186/s11671-020-03313-0

**Published:** 2020-04-21

**Authors:** Ayman Maqsood, Yaoyao Li, Juan Meng, Dandan Song, Bo Qiao, Suling Zhao, Zheng Xu

**Affiliations:** 1 Key Laboratory of Luminescence and Optical Information, Beijing Jiaotong University, Ministry of Education, Beijing, 100044 China; 2 Institute of Optoelectronics Technology, Beijing Jiaotong University, Beijing, 100044 China

**Keywords:** Mixed cations, Morphology control, Lead halide perovskite, Perovskite solar cells

## Abstract

The utilization of mixed cations is beneficial for taking the advantages of cations and achieving highly efficient perovskite solar cells (PSCs). Herein, the precisely small incorporation of CH(NH_2_)_2_ (FA) cations in methyl ammonium lead iodide (MAPbI_3_) enables the formation of compact, smooth perovskite film with high crystallinity. Consequently, the short-circuit current and the fill factor of the PSCs based on FA_*x*_MA_1-*x*_PbI_3_ perovskite are greatly improved, leading to the enhanced device efficiency. The champion PSC based on FA_0.1_MA_0.9_PbI_3_ exhibits a remarkably high efficiency of 22.02%. Furthermore, the PSCs based on FA_0.1_MA_0.9_PbI_3_ perovskite also show improved device stability. This work provides a simple approach to fabricate high-quality perovskite films and high-performance PSCs with better stability.

## Introduction

From the past decade, the increase in industrial and domestic energy needs has not only created an energy crisis but also causing problems derived from global warming. Semiconductor technology played a significant role to overcome these crises with minimal environmental problems. Semiconductor material-based solar cells are environmental friendly such as silicon, compound semiconductor, oxides, and organic materials [1, 2]. Perovskite material-based solar cells (PSCs) have become the focus due to its high power conversion efficiency rocketing from 3.8% in 2009 up to 25.2% to now [3]. The remarkable high power conversion efficiency (PCE) of PSCs relies on the unique optoelectronic properties of the perovskite materials, and perovskite material engineering is a key approach to improve the device performance.

Generally, perovskite is a material with the same type of crystal structure based on the formula *ABX*_3_, where *A* is an organic cation (like CH_3_NH_2_^+^) or alkali cation (Cs^+^) or their mixing cations, *B* is the metal anion (Pb^+2^ or Sn^+2^), and *X* refers the halide anions (*X* = *I*^−^, Br^−^, or Cl^−^) [4–10]. Among the perovskite materials, FA_*x*_MA_1-*x*_PbI_3_ (MA, methyl ammonium; FA, formamidinium) is the highly used material in PSCs. As FA^+^ has a bit larger size (ionic radius = 2.79 Å) as compared to MA^+^ (ionic radius = 2.70 Å), FA_*x*_MA_1-*x*_PbI_3_ possesses a lower band gap than MAPbI_3_, which thus allow for higher solar light harvesting efficiency [8, 11–19]. Mostly, a small amount of MA cation is doped with FA cation to fabricate FA_*x*_MA_1-*x*_PbI_3_ perovskite, which promotes the formation of the photoactive FA cation phase than pure FAPbI_3_ and lead to high device efficiency [19–21]. However, even with the incorporation of MA cations, it is yet very challenging to achieve pure black phased FA perovskite without any trace of yellow phased FA perovskite because of the larger ionic radius of FA cations specially when large amount of FA is used. This issue has been frequently observed in spite of the high efficiency of these PSCs, since this affects the structural and thermal stability of the PSCs devices [22, 23]. Therefore, in order to achieve high efficiency and device stability in FA_*x*_MA_1-*x*_PbI_3_-based PSCs, the formation of the yellow phase defects needs to be prevented. In this work, instead of using a large amount of FA cations in FA_*x*_MA_1-*x*_PbI_3_ perovskite, we use a small amount of FA to fabricate FA_0.1_MA_0.9_PbI_3_ planar film, which enables the champion device efficiency exceeding 22%. Different from the results reported from mesoporous perovskite film [24], we found the introduction of small FA into MAI within the gaps of the PbI_6_ octahedra stabilizing the MAPbI_3_ perovskite structure into a “quasi-cubic” phase at room temperature. Furthermore, the improvements here mainly derive from the largely increased *J*_SC_, benefiting from the formation of compact, smooth, high-quality perovskite film with the incorporation of FA. Furthermore, the PSCs based on FA_0.1_MA_0.9_PbI_3_ perovskite also show improved device stability.

## Results and Discussions

**Fig. 1:**
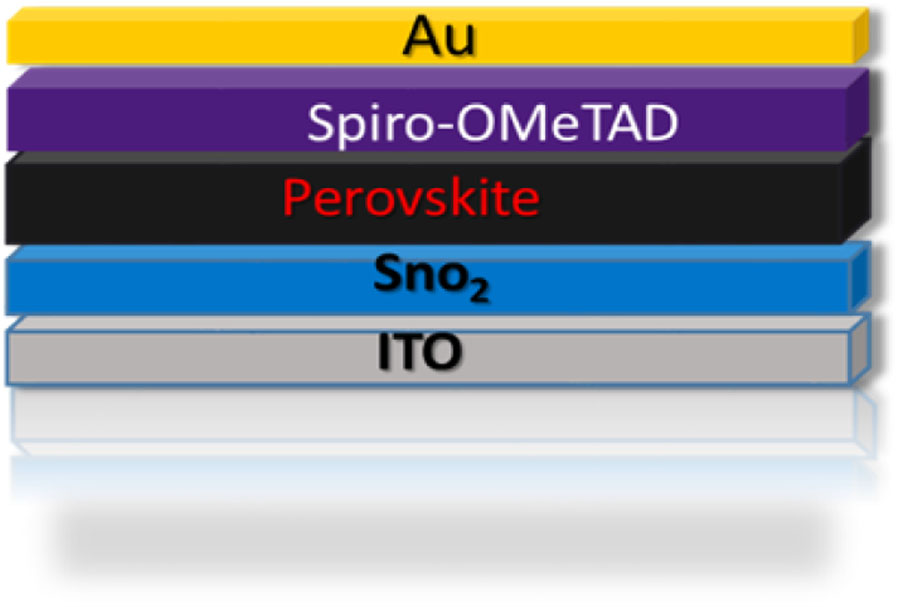
Schematic structure of perovskite solar cell

The employed device structure of the PSCs in this work is shown schematically in Fig. 1, where SnO_2_ layer is used as the electron transport layer (ETL), Spiro-OMeTAD as the hole transport layer (HTL), and gold (Au) as the anode. Both standard and modified perovskite layers were deposited on the indium tin oxide (ITO) transparent electrode as the absorbing layer by conventional one-step solvent-engineering method.

**Fig. 2:**
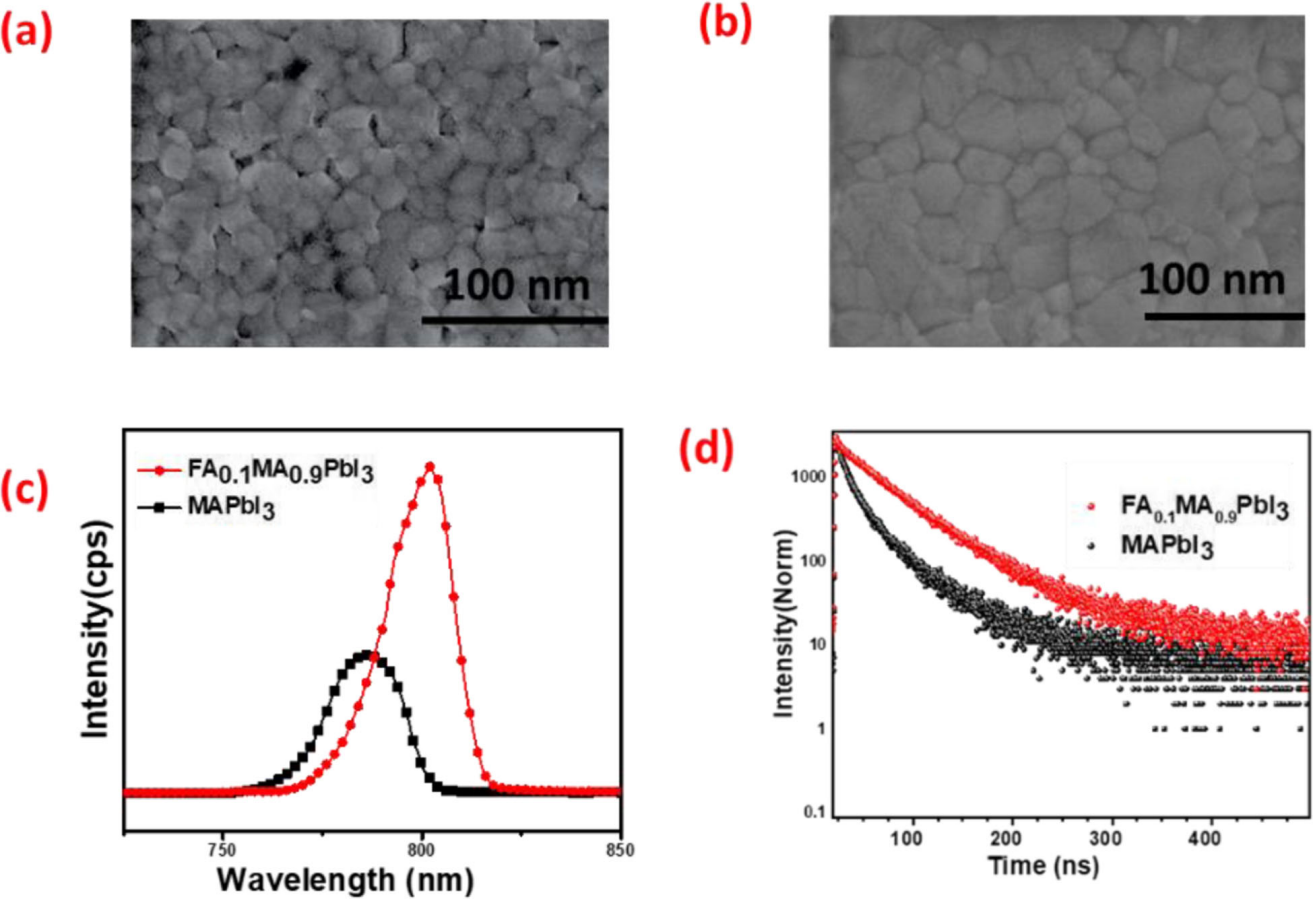
Top view SEM images of MAPbI_3_ (**a**) and FA_0.1_MA_0.9_PbI_3_ (**b**). Photoluminescence (PL) spectra of standard and modified perovskite on glass substrates. **c** Time-resolved photoluminescence (TRPL) spectra of both standard and modified perovskite films (**d**)

Scanning electron microscopy (SEM) was employed to investigate the morphology of the perovskite films. The film prepared with pristine MAPbI_3_ shows a higher ratio of grain boundaries as shown in Fig. 2a. The pinholes together with lots of grain boundaries in the perovskite film promote the non-radiative recombination and reduce the device efficiency of the PSCs. In contrast, homogenous, pinhole-free perovskite film is achieved due to the incorporation of FA cations into pristine MAPbI_3_ (FA_0.1_MA_0.9_PbI_3_) film, as shown in Fig. 2b. It presents the closely packed structure with a small enlargement in the grain size and much less grain boundaries. A compact, smooth film morphology with a large grain size will minimize the trap states and defects in the perovskite film.

The steady-state photoluminescence (PL) spectra of both MAPbI_3_ and FA_0.1_MA_0.9_PbI_3_ perovskite films are shown in Fig. 2c. As expected, a significant redshift in the emission peak is noticed. In addition, peak narrowing was also observed. This significant shift is due to the introduction of FA into MA in perovskite lattice. Furthermore, PL intensity is also increased by some extent with the addition of FA cations, which indicates the decrease in trap states and in return gives highly crystalline FA_0.1_MA_0.9_PbI_3_ film.

In order to understand more about the decrease in trap states of the modified perovskite layer as compared to a standard device, time-resolved photoluminescence (TRPL) was performed on perovskite film based on each material as shown in Fig. 2d. Since perovskite layer is deposited on the glass substrate without any transport layer, so it is expected that the carrier recombination represents only the interlayer charge transport (i.e., non-radiative recombination), which would show longer carrier lifetime and slower interlayer recombination with the incorporation of FA in MAPbI_3_ [25, 26]. Two components of time can be obtained to calculate the carrier recombination lifetime of the as-prepared perovskite film of each type by fitting the TRPL curve with bi-exponential function of time (*t*):

1 $$ F(t)={A}_1{e}^{-}\frac{t}{\tau_1}+{A}_2{e}^{-}\frac{t}{\tau_2}+{\gamma}_0 $$

where *τ*_1_ and *τ*_2_ in Eq. 1 represent the time constant of fast decay and slow decay process, respectively [27]. The fast decay *τ*_1_ component represents the surface recombination, whereas the slow decay component is related to the recombination taking place in the bulk of the perovskite structure. All the fitted TRPL parameters for standard and modified perovskite samples are summarized in table S1, and the average recombination lifetime (*τ*_ave_) of both perovskite layers was calculated approximately from the fitted curve data according to the formula shown in supplementary information. Such as, in comparison with the standard perovskite film with an average lifetime decay time of 24.61 ns, FAMAPbI_3_ (10%) film shows noticeably longer carrier average lifetime of 49.92 ns, indicating the suppression of non-radiation recombination in modified PSCs.

**Fig. 3:**
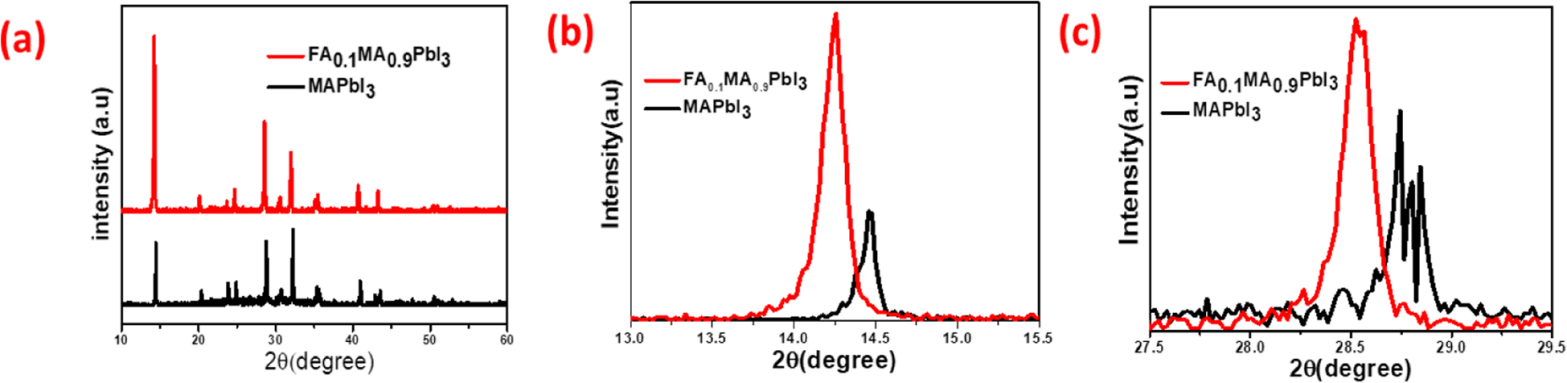
XRD patterns (**a**) for MAPbI_3_ and FA_0.1_MA_0.9_PbI_3_ perovskite films and enlarged XRD pattern of the peaks at 13–15° (**b**) and 27–29° (**c**)

X-ray diffraction (XRD) was employed to investigate the crystallinity of the perovskite films. Figure 3a shows the main diffraction peak of the perovskite film at 2*θ* of 14.24° exhibits preferred orientation with higher intensity, where the small incorporation of FA into standard MAPbI_3_ perovskite film enables the diffraction intensity much stronger, suggesting the higher crystallinity. Furthermore, the diffraction peaks of the modified perovskite layer shift towards small angle. The prominent peaks shift from 14.61° and 28.84° to 14.24° and 28.49°, respectively, as shown in Fig. 3b, c. Since the size of FA cations is larger than the MA cations, the lattice size increases with the incorporation of FA ions, which is in accordance with Bragg’s equation (2*d* sin*θ* = *nλ*). Moreover, the introduction of the FA cation with the MA also decreases the tolerance factor and provokes the formation of a stable cubic perovskite phase. Note that depending on the film morphology and deposition conditions, adding a small amount of FA (0.1) may also precede to a tetragonal phase. The full width and half maximum (FWHM) was used to estimate the grain size in the perovskite films. In FA_0.1_MA_0.9_PbI_3_, the value of FWHM of the highest peak is 0.133°, as shown in the supplementary information Fig. S1a b, which gives the evidence of increase in grain size with higher crystallinity as compared to MAPbI_3_ film (FWHM 0.174°).

**Fig. 4:**
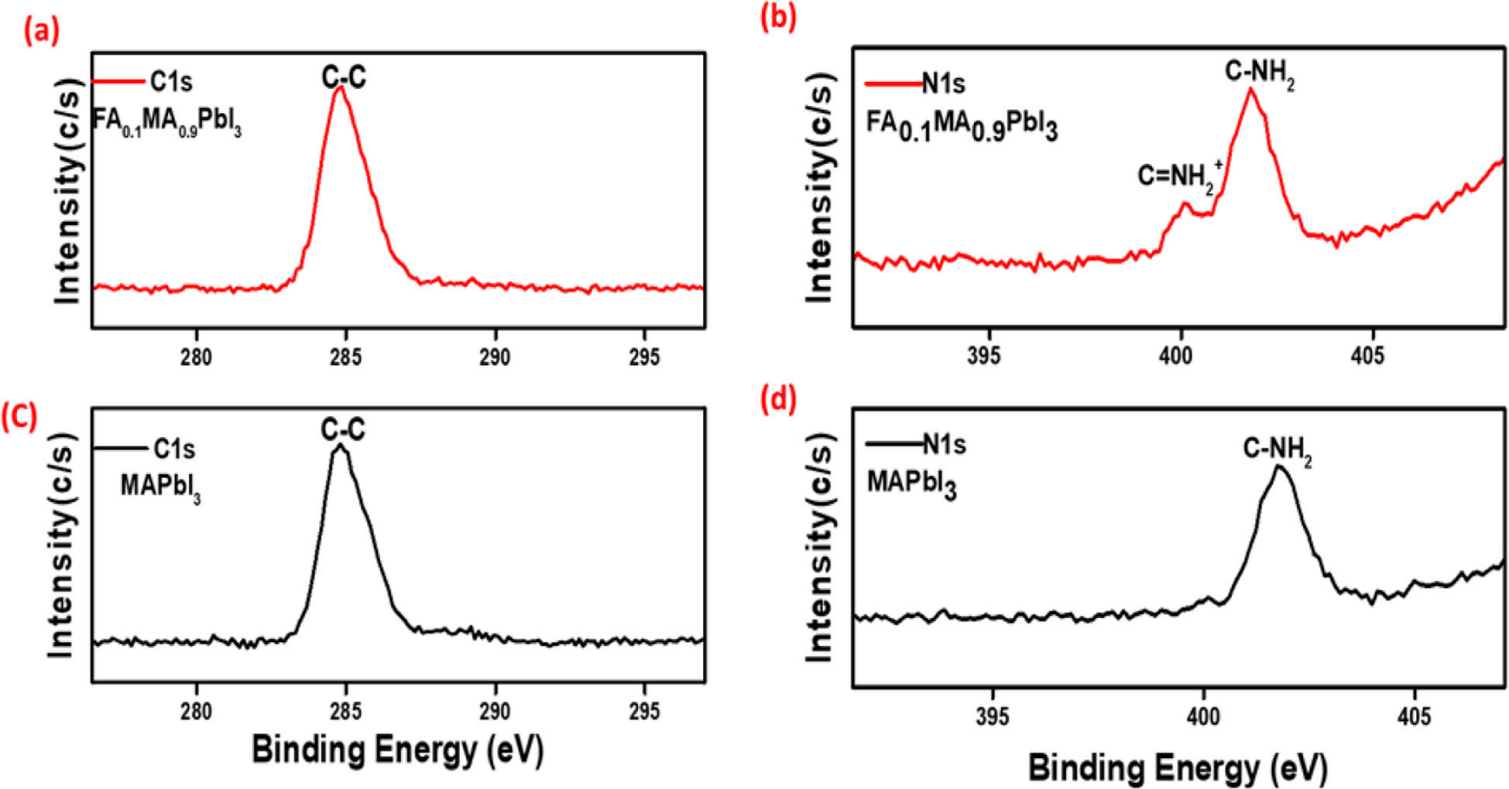
XPS measurement for elements for MAPbI_3_ and FA_0.1_MA_0.9_PbI_3_ perovskite films. Carbon (**a**, **c**). Nitrogen (**b**, **d**)

In order to confirm the element composition after precisely small incorporation of FA cations, X-ray photoelectron spectroscopy (XPS) measurements were carried out on both standard and modified perovskite layers. The presence of FA into MA cations can be confirmed by the identified C–C bond (284.8 eV) as shown in Fig. 4a, c. Furthermore, the appeared bond of C–N (401.3 eV) and C=N (400.10 eV) is from incorporated FA cations which can be observed clearly in N1s spectrum of FA_0.1_MA_0.9_PbI_3_ perovskite [27] as shown in Fig. 4b. For further analysis of the element composition, energy dispersive X-ray spectroscopy (EDX) was performed as shown in Fig S2 a & b. We can evidently equate the chemical composition; the integration of elements peaks demonstrates a quantified atomic ratio % of C to N to Pb 44.75(2.3):22.73(1.1):19.34(1) for MAPbI_3_ and 47.71 (2.3):27.34 (1.3):20.15 (1) for FA_0.1_MA0.9PbI_3_ perovskite [28].

**Fig. 5:**
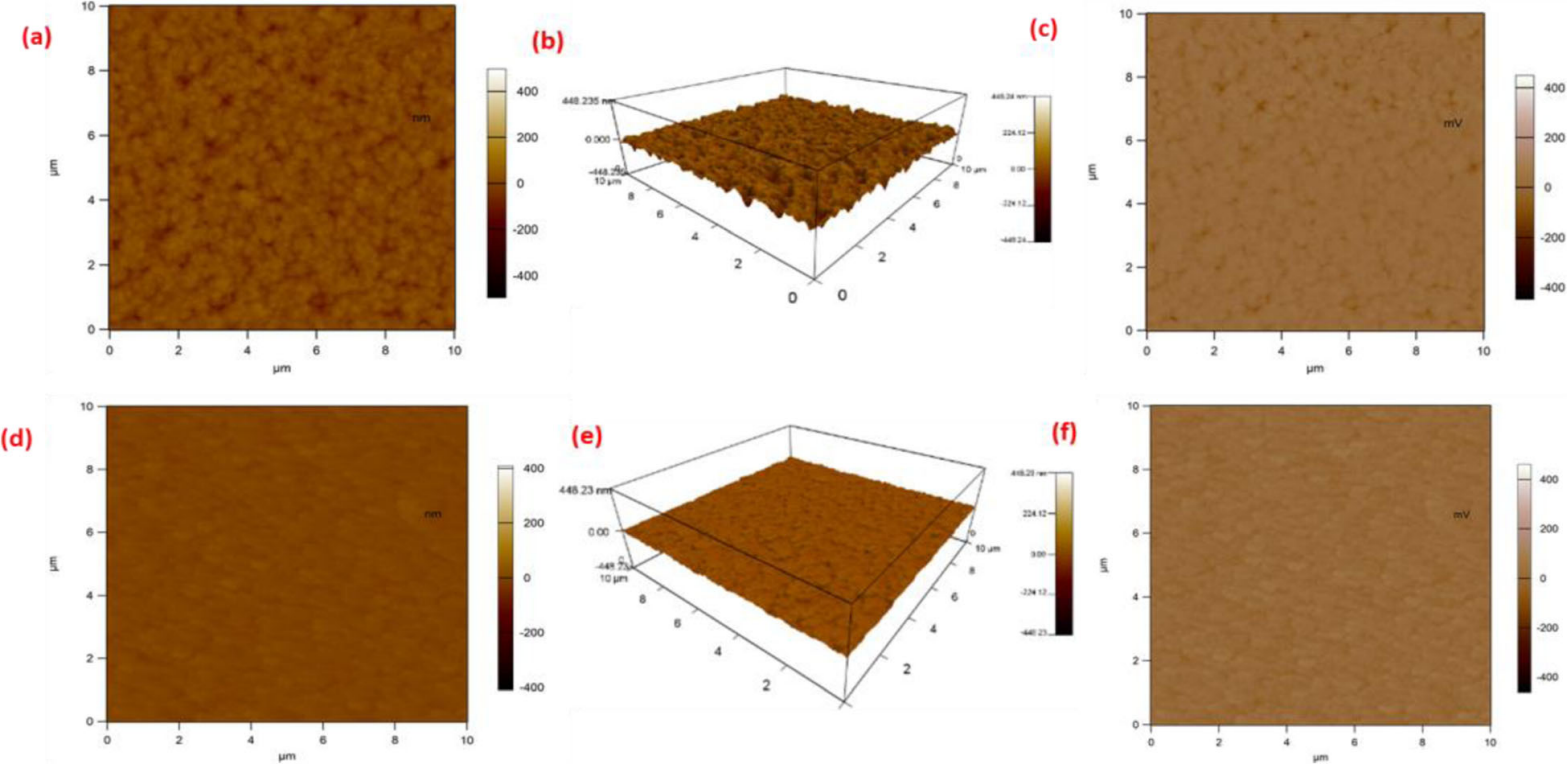
Topography images (**a**, **d**), 3D topography images (**b**, **e**), and surface potential images (**c**, **f**) of MAPbI_3_ film and FA_0.1_MA_0.9_PbI_3_ film

Kelvin probe force microscopy (KPFM) technique was used to further analyze the perovskite film, which measures the contact potential difference (CPD) between the tip and the surface of the sample beneath [14, 23]. The surface roughness is remarkably decreased from 20.488 to 4.778 nm with the incorporation of FA into MAPbI_3_ perovskite, as shown by the topographical images in Fig. 5a, d. This further reveals the compact, smooth morphology of the perovskite film with FA doping. The surface morphology of both standard and modified perovskite films in three dimensions (3D) is shown in Fig. 5b and e, respectively. The surface potential images are shown in Fig. 5c, f, and the 3D images are shown in the supplementary information Fig. S3 (b & c). It is clear that FA_0.1_MA_0.9_PbI_3_ film shows more homogenous potential distribution than MAPbI_3_ film, indicating less surface defects at the surface of FA_0.1_MA_0.9_PbI_3_ film. Meanwhile, the FA_0.1_MA_0.9_PbI_3_ film demonstrates higher surface potential at the grain boundaries than the standard perovskite film, which will lower the trapping and recombination probability of the minority carriers and stipulates current path for minority carriers to reach the corresponding selective contacts. Herein, it will improve the overall performance of the PSCs heading towards better charge transport with suppressed recombination.

**Fig. 6:**
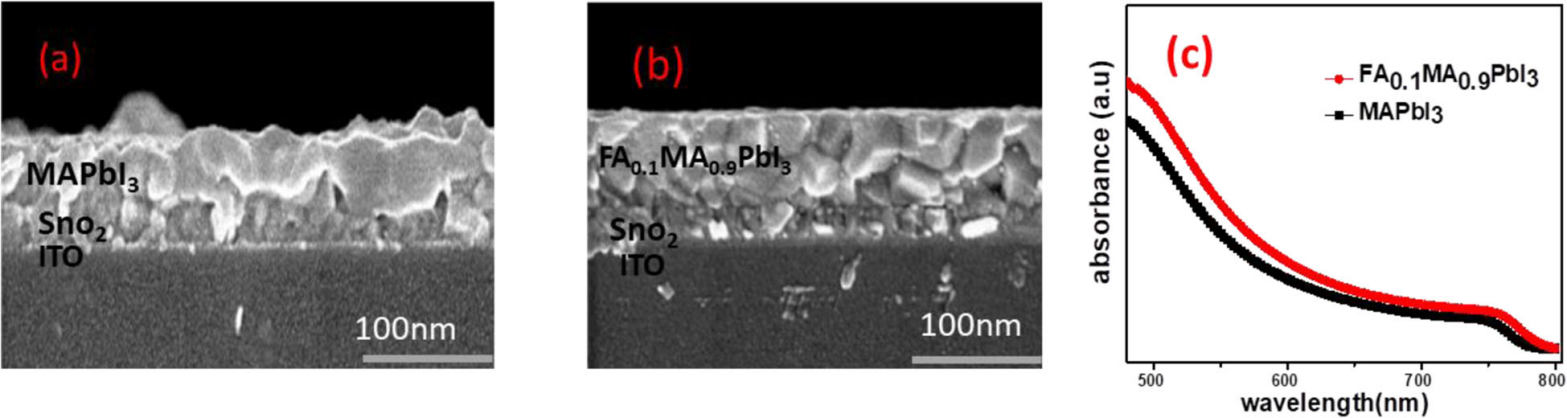
Cross-sectional images of MAPbI_3_ (**a**) and FA_0.1_MA_0.9_PbI_3_ (**b**) films on the top of ETL/ITO and the absorption spectra (**c**) of perovskite films

Cross-sectional SEM images of MAPbI_3_ film and FA_0.1_MA_0.9_PbI_3_ film are shown in Fig. 6a, b. These perovskite films are fabricated on the top of SnO_2_ ETL. It can be seen clearly that the ETL/perovskite interface is greatly improved with the incorporation of FA in perovskite. It can also be seen that FA_0.1_MA_0.9_PbI_3_ film is much more compact and smooth than MAPbI_3_ film. These improvements favor for efficient carrier extraction at interfaces. Ultraviolet-visible (UV-Vis) absorption spectra were measured to analyze the absorption features of the perovskite films, as shown in Fig. 6c. FA_0.1_MA_0.9_PbI_3_ film shows slightly higher absorption intensities than the MAPbI_3_ film. The band gap values have been calculated using the Tauc plot spectra shown in S4 a & S4b, which are 1.58 eV for MAPbI_3_ and 1.54 eV for FA_0.1_MA_0.9_PbI_3_, demonstrating that the small incorporation of FAI cations into the MAI lattice matrix reduces the band gap. The reduced band gap is beneficial to develop high-efficiency perovskite solar cells.

**Fig. 7:**
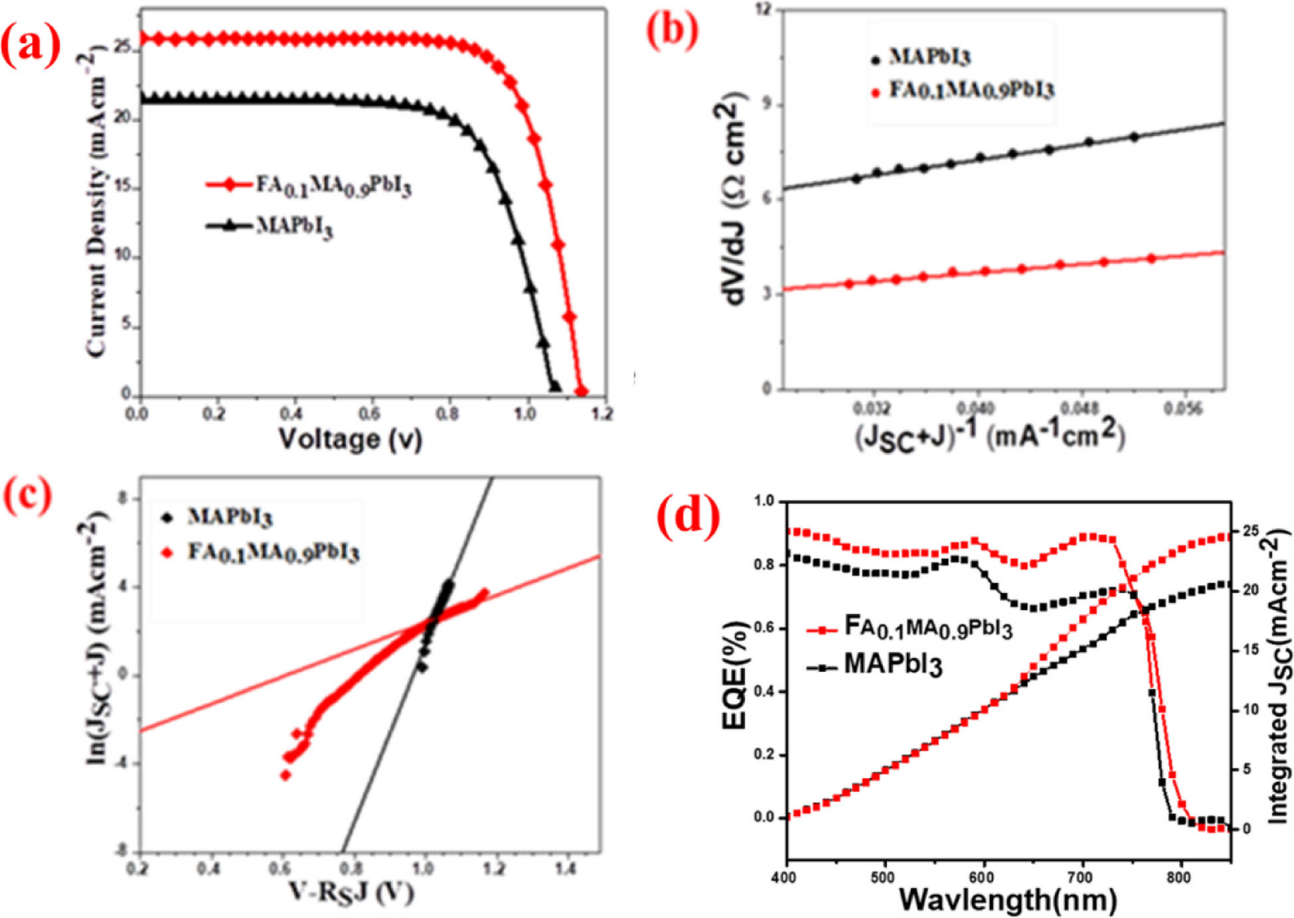
J-V curves (**a**), *dV*/*dJ* vs. (*J*_SC_ + *J*)^−1^ plots with linear fitting curve (**b**), and ln (*J*_SC_ + *J*) vs. (*V* − *R*_S_*J*) plots with linear fitting curves for the PSCs based on MAPbI_3_ and FA_0.1_MA_0.9_PbI_3_ (**c**)_._ Corresponding EQE spectra for modified perovskite in comparison with standard PSCs (**d**)

The PSCs based on MAPbI_3_ (standard PSC) and FA_0.1_MA_0.9_PbI_3_ (modified PSC) are constructed with the structure of ITO/SnO_2_/perovskite/Spiro-OMeTAD/Au. The current density-voltage (J-V) curves are shown in Fig. 7a, and the corresponding photovoltaic parameters are listed in Table 1. It is clear that the short-circuit current density (J_SC_) of modified PSCs is obviously higher than that of standard PSCs, leading to a significant increase in device efficiency. The maximum PCE of modified PSCs is 22.02% with an open-circuit voltage (*V*_OC_) of 1.13 V, *J*_SC_ of 25.87 mAcm^−2^, and a fill factor (FF) of 0.75. The remarkable improvements in *J*_SC_ and PCE of modified PSCs based on FA_0.1_MA_0.9_PbI_3_ strongly imply improved carrier collection. Due to the compact and smooth surface features of FA_0.1_MA_0.9_PbI_3_ films with a large grain size and better crystallinity, charge extraction and transport are enabled with minimal loss induced by recombination processes. Hence, *J*_SC_ is greatly increased, and, meanwhile, *V*_OC_ is also improved. The increased *J*_SC_ is also partially contributed by the reduced band gap and the enhanced absorption in FA_0.1_MA_0.9_PbI_3_ film (as revealed by the absorption features shown in Fig. 6c). To further explore the effect of FA into MAPbI_3_, we also used different ratios of FA (5–20%), and the resultant device performance of the corresponding PSCs is shown in Fig. S5 and Table S2. The incorporation of FA in the perovskite with a molar ratio from 5 to 20% increases *J*_SC_ and PCE, achieving the high efficiency of the modified PSCs. The best values of the device performance are obtained in the condition of using FA_0.1_MA_0.9_PbI_3_.

**Table 1:** Photovoltaic characteristics of standard and modified PSCs

Perovskite layer	Champion				Average			
	*V*_OC_ (V)	*J*_SC_ (mA/cm^2^)	FF	PCE (%)	*V*_OC_ (V)	*J*_SC_ (mA/cm^2^)	FF	PCE (%)
MAPbI_3_	1.07	21.48	0.71	16.22	1.07	19.09	0.70	15.06
FA_0.1_MA_0.9_PbI_3_	1.13	25.87	0.75	22.02	1.10	24.99	0.75	20.87

To investigate the underlying mechanisms for the remarkably improved device performance with the small incorporation of FA, the parameters of series resistance (*R*_S_) and reverse saturable current density (*J*_0_) have been characterized [29, 30]. The J-V characteristics are stated by:

1 $$ J={J}_{\mathrm{ph}}-{J}_0\left[\exp \left(\frac{e\left(V+ AJ{R}_S\right)}{m{K}_{\beta }T}\right)-1\right]-\frac{V+J{R}_S}{R_{\mathrm{SH}}} $$

where *J* is the current flowing through the external load, *J*_ph_ is the photocurrent density generated by a diode, *A* is the device area, *R*_SH_ is the shunt resistance, *m* refers to ideality factor of the pn juntion, *K*_*β*_ is the Boltzman’s constant, and *T* and *e* are the temperature and electronic charge, respectively [31, 32]. For an ideal condition (*R*_SH_ is large enough) [33, 34], Eq. 1 can be written as:

2 $$ \frac{dV}{dJ}=\frac{A{K}_{\beta }T}{e}{\left({J}_{\mathrm{ph}}+J\right)}^{-1}+{R}_S $$

3 $$ \ln \left({J}_{\mathrm{SC}}+J\right)=\frac{e}{A{K}_{\beta }T}\left(V-{R}_S\ J\right)+\ln {\mathrm{J}}_0 $$

*R*_S_ can be obtained from the –*dV*/*dJ* vs (*J*_SC_ − *J*)^−1^ plots in Fig. 7b with a linear fitting curve in accordance with Eq. 2, which are 4.8 Ω cm^2^ and 2.3 Ω cm^2^ for standard PSC with MAPbI_3_ and modified PSC with FA_0.1_MA_0.9_PbI_3_, respectively. This decrease in *R*_S_ for the modified PSC indicates better carrier transport and contributes to the high *J*_SC_. *J*_0_ determined from ln (*J*_SC_ + *J*) vs. (*V* − *R*_S_*J*) plots in Fig. 7c is 1.43 × 10^−2^, and 1.16 × 10^−5^ mAcm^−2^ for MAPbI_3_ and FA_0.1_MA_0.9_PbI_3_ PSCs, respectively. A smaller *J*_0_ indicates lower recombination, and thus, *V*_OC_ of the modified PSCs is improved. The reduced recombination by FA incorporation is also consitent with the KPFM measurement. Moreover, the crosssponding external quantum efficiency (EQE) has been calculated, where broad photoresponses with high values are obtained; the calculated integrated current densities (*J*_SC_) are 24.88 mAcm^−2^ and 20.25 mAcm^−2^ for best modified and standard devices, respectively, as shown in Fig. 7d which is consistent with the *J*_SC_ value calculated from J-V test.

**Fig. 8:**
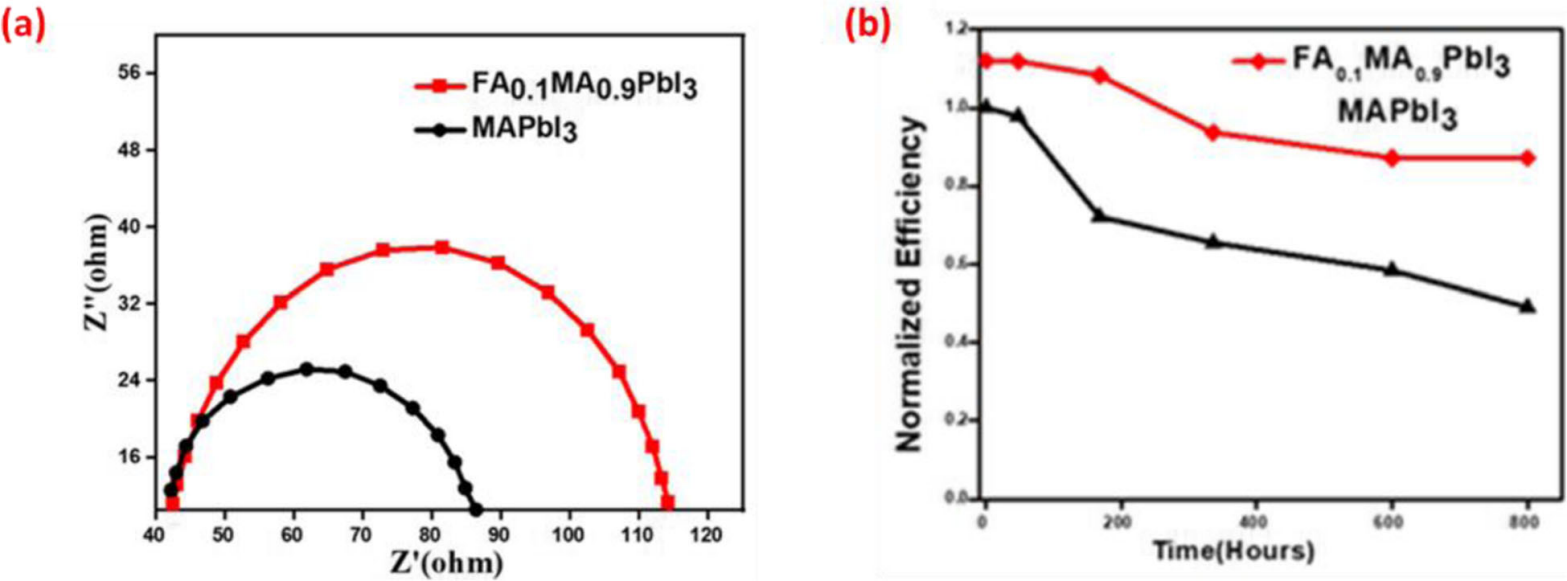
Electrochemical impedance spectroscopy (EIS) Nyquist plot for modified perovskite in comparison with standard PSCs (**a**). Normalized PCE vs. time plots of standard and modified PSCs (**b**)

In general, EIS is a suitable tool to analyze the internal electrical process of PSCs. Herein, EIS were performed as a voltage function. The obtained data were fitted with ZView using appropriate equivalent circuit as shown in Fig. 8a. The recombination resistance (*R*rec) of each perovskite solar cell is calculated from the diameter of the semicircle. It can be seen clearly that *R*rec of modified perovskite solar increased with the small incorporation of FA into MAPbI_3_ which indicates the significant decrease in undesired recombination and in return lowers the defect density of PSCs.

Figure 8b shows that the modified PSC with FA_0.1_MA_0.9_PbI_3_ keeps its original value up to 80% even after 800 h, while the standard PSC with MAPbI_3_ only retains less than 60% of its original value. The improved stability of the PSC with FA_0.1_MA_0.9_PbI_3_ correlates to the high quality of the FA_0.1_MA_0.9_PbI_3_ perovskite film. The device stability issues of the PSCs were also characterized within different time intervals under ambient conditions.

**Fig. 9:**
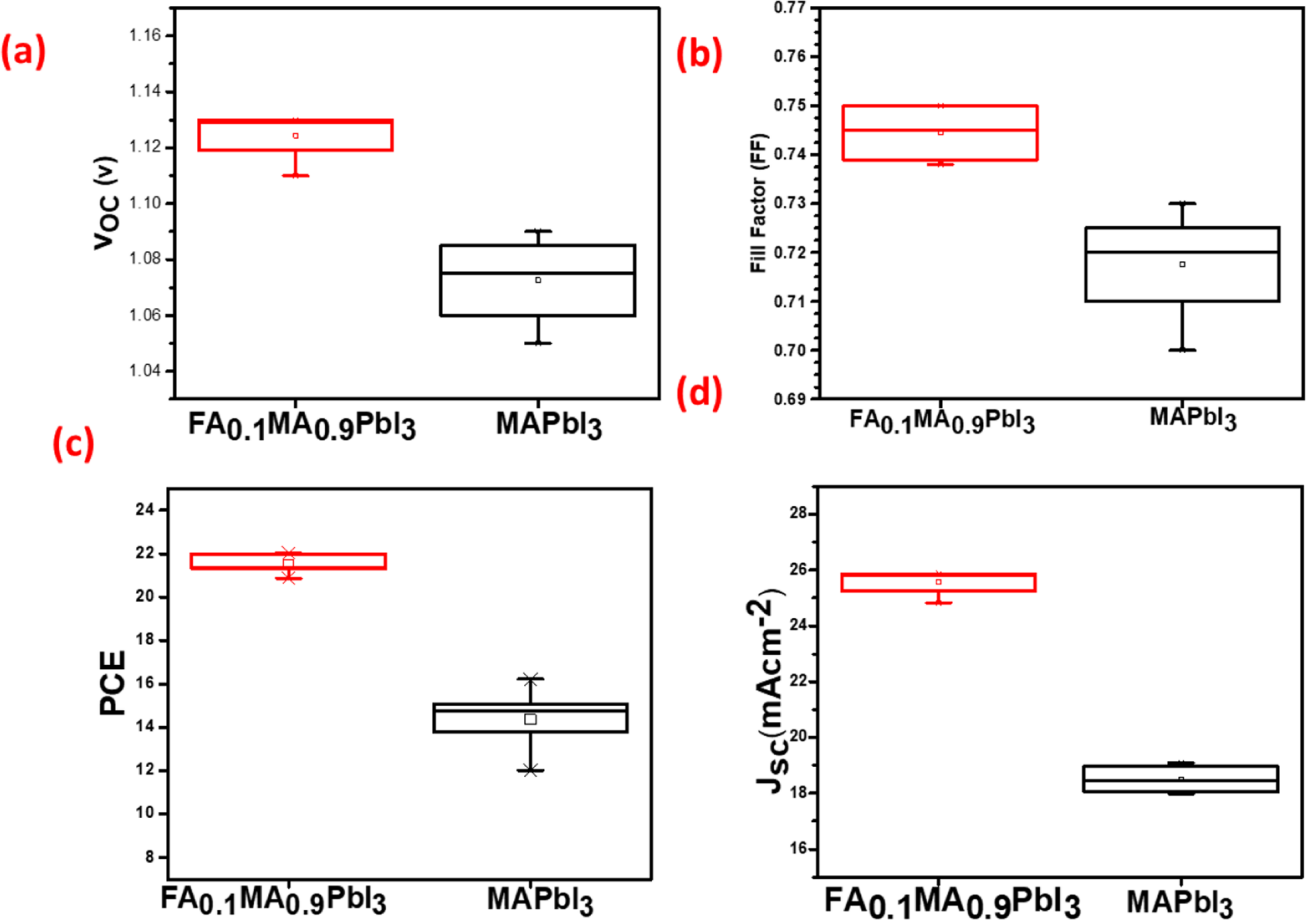
Statistics of *V*_OC_, FF PCE, and *J*_SC_ from more than 40 devices for each kind of PSCs (**a**–**d**)

In order to confirm the repeatability of the PSCs, the average performance of both MAPbI_3_ and FA_0.1_MA_0.9_PbI_3_ PSCs averaged from more than 40 devices are shown in Table 1, and the statistics of the photovoltaic parameters are shown in Fig. 9a–d. It can be seen that in PSCs with FA_0.1_MA_0.9_PbI_3_, the average performance is also obviously superior to the PSCs with MAPbI_3_ and show better reproducibility.

## Experimental Details

### Materials and Methods

CH_3_NH_3_I, PbI_2_, CH (NH_2_)_2_, and Spiro-OMeTAD were purchased from Xi’an Polymer Light Technology Corp. SnO_2_ was purchased from Alfa Aesar. The precursor solution of FA_*x*_MA_1-*x*_PbI_3_ was composed of PbI_2_, CH_3_NH_3_I, and CH (NH_2_)_2_ stirred in a mixture of dimethyl formamide (DMF) to dimethyl sulfoxide (DMSO) (9:1, vol/vol) overnight. The concentration of FA_0.1_MA_0.9_PbI_3_ precursor solution was 0.3 mol/ml. The indium tin oxide (ITO)-coated substrates has a sheet resistance of 15 Ω/□. All materials were used directly without any further purification.

### Device Fabrication

All the PSCs were fabricated on ITO glass substrates. SnO_2_ was deposited on the pre-cleaned ITO substrate as an electron transport layer (ETL). Perovskite layers were deposited in the N_2_-filled glove box at 5000 rpm for 35 s. At 29 s before the spin coating stop, antisolvent toluene was dropped onto the substrate. After that, the substrate was transferred to the hot palate for annealing for 8 min at 80 °C and then for 10 min at 120 °C. After cooling down, the hole transport material Spiro-OMeTAD was deposited on the top of the perovskite layer by spin coating at 3000 rpm for 30 s. After the spin coating of all the layers was finished, the samples were kept out of the glove box overnight for a better oxidation process. Finally, 80 nm of gold (Au) was deposited by the thermal evaporation under 4 × 10^−4^ Pa vacuum conditions to complete the device structure.

### Device Characterization

Current-voltage characterization was carried out with a digital source meter (Keithley Model 2400) at AM 1.5G at 100 mW cm^−2^. Scanning electron microscope (SEM) measurements were made by 4800. X-ray diffraction (XRD) patterns were collected with a D/max 2200 V X-ray powder diffractometer with Cu Kα radiation (*λ* = 1.540 A). Kelvin probe force microscopy (KPFM) measurements were taken using the MFP-3D infinity of Asylum Research. All characterizations were accomplished under constant exposure to ambient conditions and without device encapsulation.

## Conclusion

In our work, a precisely small amount of FA cations is introduced into MA cations of standard MAPbI_3_-based perovskite film to enhance the film quality in terms of smoothness and crystallinity with full surface coverage. The remarkable PCE of 22.02% and significantly enhanced *J*_SC_ has been obtained from the PSCs based on FA_0.1_MA_0.9_PbI_3_ perovskite. Furthermore, the enhancement in *V*_OC_ as a consequence of reduced carrier recombination is also obtained. These results reveal that high-efficiency PSCs with superior stability can be repeatability fabricated based on the compact, smooth perovskite film with improved crystallinity enabled by the incorporation of a small value of FAI cations into MAPbI_3_.

## Supplementary information

**Additional file 1.**

## Data Availability

The datasets supporting the results of this article are included within the article.

## **A**bbreviat**ions**

PSC: Perovskite solar cell
SEM: Scanning electron microscopy
XRD: X-ray diffraction
FWHM: Full width and half maximum
PL: Photoluminescence
TRPL: Time-resolved photoluminescence
KPFM: Kelvin probe force microscopy
EIS: Electrochemical impedance spectroscopy
