# Supplementary material for: Perovskite Solar Cells Based on Compact, Smooth FA0.1MA0.9PbI3 Film with Efficiency Exceeding 22%
Source: Nanoscale Res Lett. 2020 Apr 21;15:89. doi: 10.1186/s11671-020-03313-0 (PMC7174521; doi:10.1186/s11671-020-03313-0)
Supplement: Supplementary file 1 — Additional file 1. [file 11671_2020_3313_MOESM1_ESM.pdf]

Supporting information

Compact, Smooth $FA_{0.1}MA_{0.9}PbI_3$ perovskite film with improved crystallinity for perovskite solar cells with efficiency exceeding 22%

Ayman Maqsood, [ab] *Zheng Xu,* *[ab] *Dandan Song,* [ab] *Bo Qiao,* [ab] *Juan Meng,* [ab] *Yaoyao Li,* [ab] *Suling Zhao*

1. Key Laboratory of Luminescence and Optical Information (Beijing Jiaotong University), Ministry of Education, Beijing 100044, China

2. Institute of Optoelectronics Technology, Beijing Jiaotong University, Beijing 100044, China

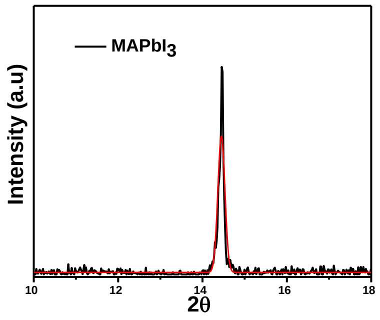

Fig S1(a*): The full width and half maximum (FWHM) of $MAPbI_3$ based perovksite solar cell*

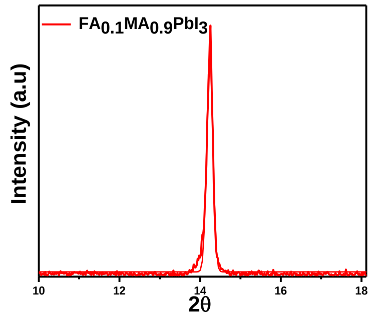

Fig S1(b): *The full width and half maximum (FWHM) of $FA_{0.1}MA_{0.9}PbI_3$ based perovksite solar cell*

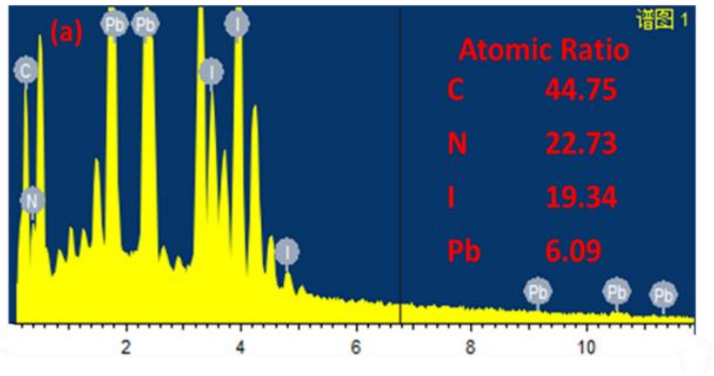

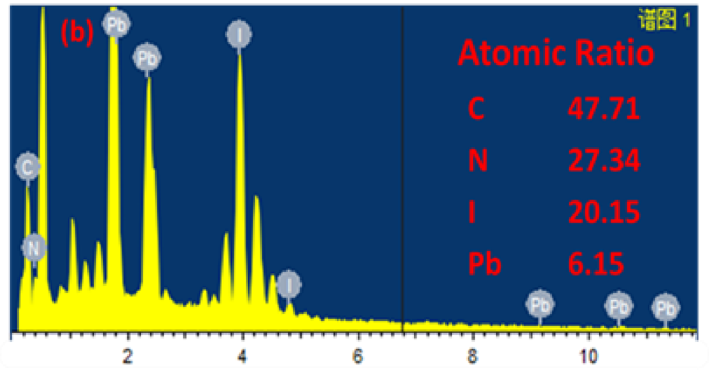

Fig S2: *EDX measurement MAPbI$_3$(a) FAMAPbI$_3$ (b)*

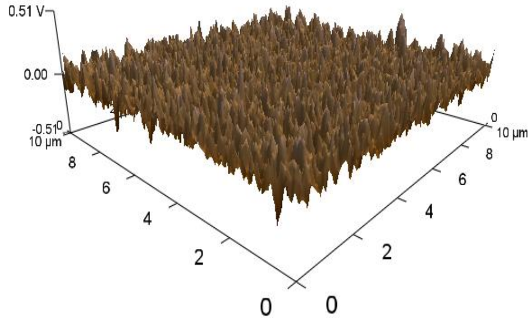

Fig S3 *(a):* Kelvin probe force microscopy (KPFM) images*: 3D view of surface potential of MAPbI$_3$ based perovksite layer*

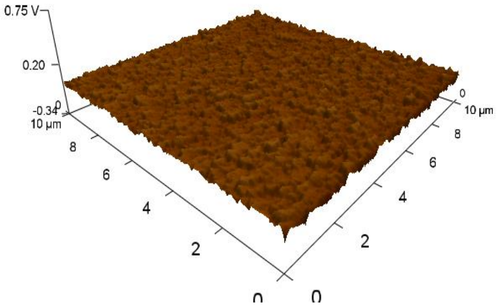

Fig S3 *(b):* Kelvin probe force microscopy (KPFM) images*: 3D view of surface potential of FA$_{0.1}$MA$_{0.9}$PbI$_3$ based perovksite layer*

Table S1. Parameters of the TRPL spectroscopy of standard film and modified (10%) film on the glass substrate.

The average lifetime (τ_{ave}) is calculated using:

$$\tau_{ave} = (A_1\tau_1^2 + A_2\tau_2^2)/(A_1\tau_1 + A_2\tau_2)$$

samples	τ_1 (ns)	A_1	τ_2 (ns)	A_2
**Standard film**	10.76	1104.48	34.06	511.93
**modified (10%)**	29.39	1164.96	58.32	1433.82

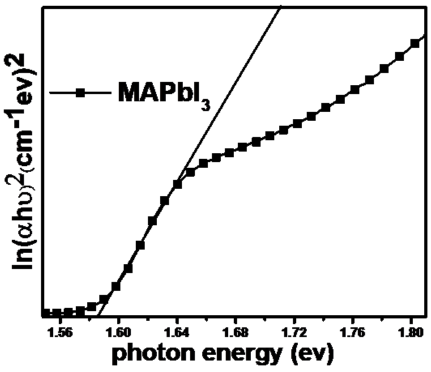

Fig S4 (a): *The Tauc plot to estimated band gap value for $MAPbI_3$*

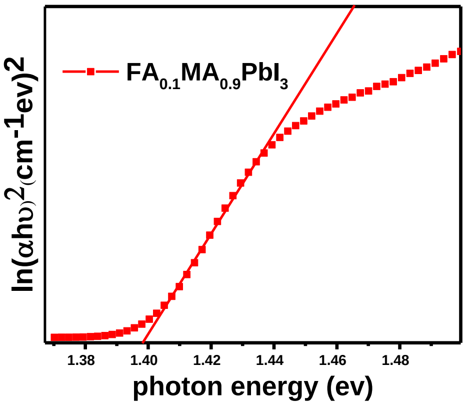

Fig S4 (b): *The Tauc plot to estimated band gap value for $FA_{0.1}MA_{0.9}PbI_3$*

***Table S2**: Photovoltaic characteristics with different concentration of FAI into standard $MAPbI_3$ PSCs.*

Perovskite layer	VOC [V]	J_{SC} [$mAcm^{-2}$]	FF	PCE %
$FA_{0.5}MA_{0.95}PbI_3$	1.08	23.47	0.72	18.22
$FA_{0.15}MA_{0.85}PbI_3$	1.08	25.38	0.73	19.97
$FA_{0.2}MA_{0.80}PbI_3$	1.07	24.77	0.71	18.66

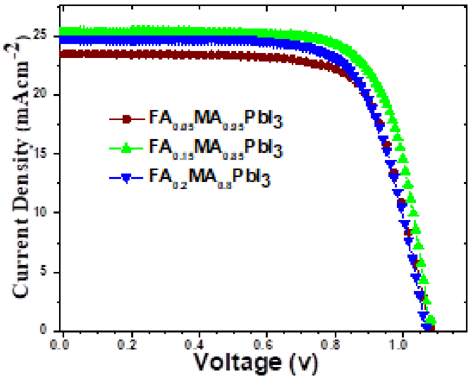

***Fig S5:** J-V with different concentration of FAI into standard $MAPbI_3$ PSCs.*

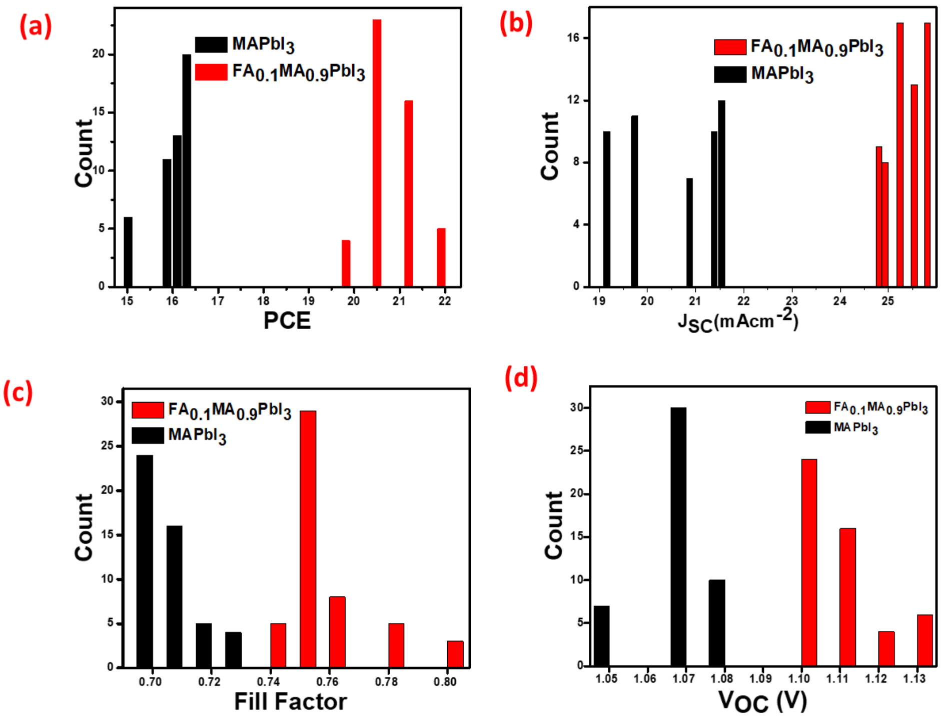

***Fig S6:** histogram of performance over 40 devices of PCE, Jsc, FF and V_{OC} of standard and modified PSC.*
